# (*R*)-2,2′-Bis(meth­oxy­meth­oxy)-1,1′-binaphth­yl

**DOI:** 10.1107/S1600536812014699

**Published:** 2012-04-13

**Authors:** Liang Zhou, Wumanjiang Eli

**Affiliations:** a The Key Laboratory of Applied Catalysis, Xinjiang Technical Institute of Physics and Chemistry, Chinese Academy of Sciences, Urumqi, Xinjiang 830011, People’s Republic of China

## Abstract

The asymmetric unit of the title compound, C_24_H_22_O_4_, contains two independent mol­ecules in both of which the naphthalene ring systems adopts a *transoid* arrangement. The dihedral angles between the naphthalene ring system in the two mol­ecules are 83.0 (1) and 89.0 (1)°. There are slight differences in the C(H_3_)—O—C(H_2_)—O– torsion angles of the eqivalent meth­oxy­meth­oxy groups. In the crystal, weak C—H⋯O hydrogen bonds are present.

## Related literature

For general background to the application of 1,1′-binaphthol (BINOL) derivatives in asymmetric synthesis, see: Brunel *et al.* (2006 ▶). For the synthesis, see: Shi & Wang (2002 ▶). For related structures, see: Tachi *et al.* (1999 ▶); Zong *et al.* (2011 ▶).
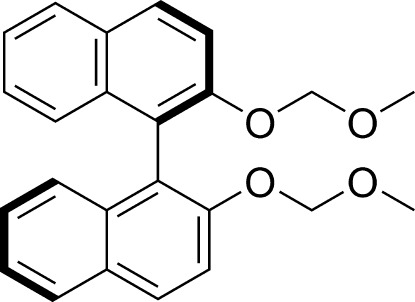


## Experimental

### 

#### Crystal data

C_24_H_22_O_4_
*M* *_r_* = 374.42
Orthorhombic, 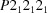

*a* = 10.8608 (13) Å
*b* = 12.6158 (14) Å
*c* = 29.419 (3) Å
*V* = 4030.9 (8) Å^3^
*Z* = 8
Mo *K*α radiation
μ = 0.08 mm^−1^
*T* = 296 K
0.20 × 0.20 × 0.18 mm

#### Data collection

Bruker APEXII CCD diffractometer
Absorption correction: multi-scan (*SADABS*; Bruker, 2004 ▶) *T*_min_ = 0.984, *T*_max_ = 0.985
19981 measured reflections
5112 independent reflections
3899 reflections with *I* > 2σ(*I*)
*R*_int_ = 0.029

#### Refinement

*R*[*F* ^2^ > 2σ(*F* ^2^)] = 0.061
*wR*(*F* ^2^) = 0.149
*S* = 1.01
5112 reflections
509 parameters
H-atom parameters constrained
Δρ_max_ = 0.17 e Å^−3^
Δρ_min_ = −0.16 e Å^−3^

### 

Data collection: *APEX2* (Bruker, 2004 ▶); cell refinement: *SAINT* (Bruker, 2004 ▶); data reduction: *SAINT*; program(s) used to solve structure: *SHELXS97* (Sheldrick, 2008 ▶); program(s) used to refine structure: *SHELXL97* (Sheldrick, 2008 ▶); molecular graphics: *SHELXTL* (Sheldrick, 2008 ▶); software used to prepare material for publication: *publCIF* (Westrip, 2010 ▶).

## Supplementary Material

Crystal structure: contains datablock(s) global, I. DOI: 10.1107/S1600536812014699/lh5447sup1.cif

Structure factors: contains datablock(s) I. DOI: 10.1107/S1600536812014699/lh5447Isup2.hkl

Additional supplementary materials:  crystallographic information; 3D view; checkCIF report

## Figures and Tables

**Table 1:** Selected torsion angles (°)

C1—O1—C2—O2	−68.1 (6)
C24—O4—C23—O3	64.4 (7)
C25—O5—C26—O6	−73.6 (5)
C48—O8—C47—O7	65.8 (7)

**Table 2:** Hydrogen-bond geometry (Å, °)

*D*—H⋯*A*	*D*—H	H⋯*A*	*D*⋯*A*	*D*—H⋯*A*
C34—H34*A*⋯O8^i^	0.93	2.40	3.251 (5)	152
C39—H39*A*⋯O5^ii^	0.93	2.52	3.417 (5)	161

Symmetry codes: (i) 
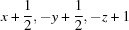
; (ii) 
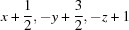
.

**Fig. 1.**
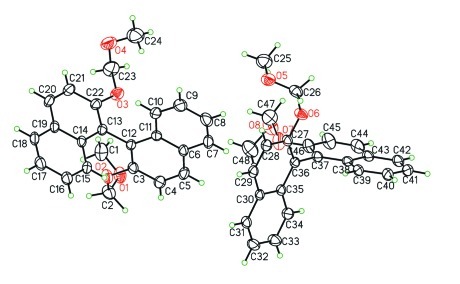
The molecular structure of (I), showing displacement ellipsoids at the 50% probability level.

**Fig. 2.**
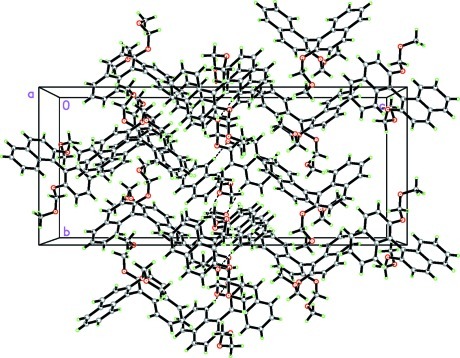
Part of the crystal structure showing hydrogen bonds as dashes lines.

## supplementary crystallographic information

### Comment

Optically active 1,1'-binaphthol(BINOL) and its derivatives are one of the most important chiral ligands, and have been widely used for in asymmetric synthesis for many years (Brunel *et al.* 2006). Although 2,2'-bis(methoxymethoxy)-1,1'-binaphthyl is a very important intermediate to many chiral ligands for various metal complex catalysis, none of its structural properties have been explored. Within our ongoing project of synthesizing BINOL derivatives, we have synthesized the title compound determined its crystal structure. Examples of similar structures have been published (Tachi *et al.*, 1999; Zong *et al.*, 2011).

The asymmetric unit of the title compound is shown in Fig. 1. There are two crystallographically independent molecules in which the two naphthalene ring systems in each, adopt a *transoid* arrangement. There are only slight differences in the torsion angles of the eqivalent methoxymethoxy groups (Table 1). In the crystal, Fig. 2, molecules are linked by weak C—H···O hydrogen bonds.

### Experimental

The title compound (I) was synthesized from (*R*)-BINOL according to the literature method (Shi & Wang, 2002). Under a nitrogen atmosphere, (*R*)-BINOL (5.72 g, 20 mmol) was added to a suspension of NaH (2.40 g, 100 mmol) in anhydrous THF (40 ml) at 273K with stirring. The resulting solution was further stirred at 273K for 10 min, and then methoxymethyl chloride (3.65 ml, 48 mmol) was slowly added. The mixture was allowed to warm to room temperature and stirred for 4 h. After the standard procedures of quenching, washing and drying the organic layers, the solvent was removed. A crystal suitable for X-ray analysis was grown from a solution of (I) in ethyl acetate and petroleum ether by slow evaporation at room temperature.

### Refinement

Hydrogen atoms were placed in calculated positions with C—H = 0.97 Å (methylene), 0.96 Å (methyl) and 0.93 Å (aromatic) with *U*_iso_(H) = 1.2U_eq_(C) (methylene C and aromatic C) or 1.5U_eq_(C) (methyl C). In the absence of significant anomalous dispersion effects Friedel pairs were measured. The absolute configuration is known from the starting material.

### Crystal data

**Table:**

C_24_H_22_O_4_	*F*(000) = 1584
*M**_r_* = 374.42	*D*_x_ = 1.234 Mg m^−^^3^
Orthorhombic, *P*2_1_2_1_2_1_	Mo *K*α radiation, λ = 0.71073 Å
Hall symbol: P 2ac 2ab	Cell parameters from 9472 reflections
*a* = 10.8608 (13) Å	θ = 2.5–27.3°
*b* = 12.6158 (14) Å	µ = 0.08 mm^−^^1^
*c* = 29.419 (3) Å	*T* = 296 K
*V* = 4030.9 (8) Å^3^	Block, colourless
*Z* = 8	0.20 × 0.20 × 0.18 mm

### Data collection

**Table:**

Bruker APEXII CCD diffractometer	5112 independent reflections
Radiation source: fine-focus sealed tube	3899 reflections with *I* > 2σ(*I*)
Graphite monochromator	*R*_int_ = 0.029
φ and ω scans	θ_max_ = 27.5°, θ_min_ = 1.8°
Absorption correction: multi-scan (*SADABS*; Bruker, 2004)	*h* = −12→14
*T*_min_ = 0.984, *T*_max_ = 0.985	*k* = −15→16
19981 measured reflections	*l* = −38→32

### Refinement

**Table:**

Refinement on *F*^2^	Primary atom site location: structure-invariant direct methods
Least-squares matrix: full	Secondary atom site location: difference Fourier map
*R*[*F*^2^ > 2σ(*F*^2^)] = 0.061	Hydrogen site location: inferred from neighbouring sites
*wR*(*F*^2^) = 0.149	H-atom parameters constrained
*S* = 1.01	*w* = 1/[σ^2^(*F*_o_^2^) + (0.046*P*)^2^ + 1.9*P*] where *P* = (*F*_o_^2^ + 2*F*_c_^2^)/3
5112 reflections	(Δ/σ)_max_ < 0.001
509 parameters	Δρ_max_ = 0.17 e Å^−^^3^
0 restraints	Δρ_min_ = −0.16 e Å^−^^3^

### Special details

**Table:**

Geometry. All e.s.d.'s (except the e.s.d. in the dihedral angle between two l.s. planes) are estimated using the full covariance matrix. The cell e.s.d.'s are taken into account individually in the estimation of e.s.d.'s in distances, angles and torsion angles; correlations between e.s.d.'s in cell parameters are only used when they are defined by crystal symmetry. An approximate (isotropic) treatment of cell e.s.d.'s is used for estimating e.s.d.'s involving l.s. planes.
Refinement. Refinement of *F*^2^ against ALL reflections. The weighted *R*-factor *wR* and goodness of fit *S* are based on *F*^2^, conventional *R*-factors *R* are based on *F*, with *F* set to zero for negative *F*^2^. The threshold expression of *F*^2^ > σ(*F*^2^) is used only for calculating *R*-factors(gt) *etc*. and is not relevant to the choice of reflections for refinement. *R*-factors based on *F*^2^ are statistically about twice as large as those based on *F*, and *R*- factors based on ALL data will be even larger.

### Fractional atomic coordinates and isotropic or equivalent isotropic displacement parameters (Å^2^)

**Table:**

	*x*	*y*	*z*	*U*_iso_*/*U*_eq_	
O1	0.4117 (4)	0.0210 (3)	0.75572 (11)	0.0945 (10)	
O2	0.3955 (3)	0.1954 (2)	0.78134 (9)	0.0813 (9)	
O3	0.0860 (3)	0.2176 (3)	0.72124 (11)	0.0832 (9)	
O4	−0.1250 (4)	0.2162 (4)	0.70515 (17)	0.1260 (15)	
C1	0.2928 (6)	−0.0116 (5)	0.7687 (2)	0.120 (2)	
H1A	0.2664	−0.0687	0.7495	0.179*	
H1B	0.2367	0.0468	0.7658	0.179*	
H1C	0.2944	−0.0352	0.7998	0.179*	
C2	0.4595 (5)	0.0971 (4)	0.78456 (17)	0.0942 (16)	
H2A	0.5456	0.1084	0.7773	0.113*	
H2B	0.4549	0.0715	0.8156	0.113*	
C3	0.3998 (4)	0.2504 (3)	0.74082 (13)	0.0626 (10)	
C4	0.4970 (4)	0.2400 (4)	0.70941 (15)	0.0774 (12)	
H4A	0.5602	0.1919	0.7149	0.093*	
C5	0.4984 (4)	0.2999 (4)	0.67137 (14)	0.0750 (12)	
H5A	0.5624	0.2914	0.6507	0.090*	
C6	0.4067 (4)	0.3741 (3)	0.66208 (12)	0.0639 (10)	
C7	0.4095 (5)	0.4410 (4)	0.62319 (14)	0.0844 (14)	
H7A	0.4737	0.4349	0.6025	0.101*	
C8	0.3192 (6)	0.5137 (5)	0.61598 (17)	0.0992 (17)	
H8A	0.3222	0.5566	0.5903	0.119*	
C9	0.2226 (5)	0.5248 (5)	0.64653 (18)	0.0982 (17)	
H9A	0.1616	0.5750	0.6412	0.118*	
C10	0.2166 (4)	0.4622 (4)	0.68445 (14)	0.0739 (11)	
H10A	0.1514	0.4704	0.7046	0.089*	
C11	0.3077 (3)	0.3855 (3)	0.69335 (12)	0.0562 (8)	
C12	0.3056 (3)	0.3207 (3)	0.73325 (11)	0.0516 (8)	
C13	0.2020 (3)	0.3309 (3)	0.76685 (12)	0.0534 (8)	
C14	0.2173 (3)	0.3964 (3)	0.80585 (11)	0.0530 (8)	
C15	0.3265 (4)	0.4533 (3)	0.81477 (12)	0.0623 (10)	
H15A	0.3922	0.4478	0.7946	0.075*	
C16	0.3377 (4)	0.5159 (4)	0.85211 (13)	0.0780 (12)	
H16A	0.4109	0.5520	0.8575	0.094*	
C17	0.2384 (5)	0.5263 (4)	0.88279 (15)	0.0838 (14)	
H17A	0.2460	0.5701	0.9081	0.101*	
C18	0.1326 (5)	0.4728 (4)	0.87558 (13)	0.0763 (13)	
H18A	0.0682	0.4797	0.8962	0.092*	
C19	0.1179 (4)	0.4065 (3)	0.83725 (12)	0.0600 (9)	
C20	0.0081 (4)	0.3500 (4)	0.82853 (15)	0.0770 (12)	
H20A	−0.0572	0.3558	0.8488	0.092*	
C21	−0.0045 (4)	0.2878 (4)	0.79143 (16)	0.0792 (12)	
H21A	−0.0775	0.2509	0.7866	0.095*	
C22	0.0937 (4)	0.2788 (3)	0.75972 (14)	0.0635 (9)	
C23	−0.0202 (6)	0.1552 (5)	0.7137 (2)	0.1127 (19)	
H23A	−0.0056	0.1087	0.6880	0.135*	
H23B	−0.0349	0.1112	0.7402	0.135*	
C24	−0.1165 (7)	0.2764 (6)	0.6640 (3)	0.141 (3)	
H24A	−0.1962	0.3039	0.6563	0.211*	
H24B	−0.0876	0.2315	0.6399	0.211*	
H24C	−0.0599	0.3341	0.6682	0.211*	
O5	0.2538 (3)	0.6716 (3)	0.50914 (12)	0.0909 (10)	
O6	0.4394 (3)	0.6111 (2)	0.47870 (9)	0.0783 (9)	
O7	0.5160 (4)	0.3566 (2)	0.51724 (11)	0.1022 (12)	
O8	0.4472 (4)	0.1847 (3)	0.51517 (14)	0.1148 (14)	
C25	0.1766 (6)	0.5825 (5)	0.5021 (2)	0.118 (2)	
H25A	0.1165	0.5789	0.5260	0.177*	
H25B	0.2255	0.5190	0.5023	0.177*	
H25C	0.1357	0.5891	0.4734	0.177*	
C26	0.3418 (4)	0.6854 (4)	0.47605 (16)	0.0817 (13)	
H26A	0.3756	0.7563	0.4786	0.098*	
H26B	0.3031	0.6795	0.4465	0.098*	
C27	0.5225 (4)	0.6199 (3)	0.51385 (12)	0.0563 (9)	
C28	0.5085 (4)	0.6944 (3)	0.54955 (12)	0.0615 (9)	
H28A	0.4421	0.7409	0.5495	0.074*	
C29	0.5917 (4)	0.6978 (3)	0.58372 (12)	0.0571 (9)	
H29A	0.5812	0.7468	0.6070	0.069*	
C30	0.6929 (3)	0.6295 (3)	0.58500 (11)	0.0505 (8)	
C31	0.7808 (4)	0.6324 (3)	0.61994 (12)	0.0648 (10)	
H31A	0.7705	0.6799	0.6438	0.078*	
C32	0.8801 (4)	0.5679 (4)	0.61975 (15)	0.0841 (14)	
H32A	0.9375	0.5714	0.6431	0.101*	
C33	0.8960 (4)	0.4950 (4)	0.58375 (15)	0.0868 (14)	
H33A	0.9645	0.4508	0.5835	0.104*	
C34	0.8121 (4)	0.4888 (3)	0.54953 (14)	0.0682 (10)	
H34A	0.8237	0.4397	0.5263	0.082*	
C35	0.7081 (3)	0.5553 (3)	0.54859 (11)	0.0498 (8)	
C36	0.6203 (3)	0.5517 (3)	0.51278 (11)	0.0493 (8)	
C37	0.6325 (3)	0.4750 (3)	0.47417 (12)	0.0550 (8)	
C38	0.6962 (3)	0.5012 (3)	0.43387 (12)	0.0604 (10)	
C39	0.7466 (4)	0.6026 (4)	0.42678 (13)	0.0717 (11)	
H39A	0.7384	0.6540	0.4492	0.086*	
C40	0.8078 (4)	0.6271 (5)	0.38709 (14)	0.0926 (16)	
H40A	0.8396	0.6948	0.3828	0.111*	
C41	0.8224 (4)	0.5502 (6)	0.35312 (16)	0.103 (2)	
H41A	0.8664	0.5664	0.3269	0.124*	
C42	0.7729 (5)	0.4525 (5)	0.35830 (15)	0.0939 (18)	
H42A	0.7815	0.4028	0.3352	0.113*	
C43	0.7083 (4)	0.4247 (4)	0.39834 (13)	0.0733 (12)	
C44	0.6552 (5)	0.3250 (4)	0.40443 (16)	0.0904 (16)	
H44A	0.6635	0.2744	0.3817	0.109*	
C45	0.5919 (6)	0.2999 (4)	0.44260 (17)	0.0960 (16)	
H45A	0.5573	0.2329	0.4460	0.115*	
C46	0.5789 (5)	0.3777 (3)	0.47759 (14)	0.0759 (12)	
C47	0.4089 (6)	0.2867 (4)	0.5151 (2)	0.1091 (18)	
H47A	0.3557	0.2995	0.5410	0.131*	
H47B	0.3623	0.3010	0.4876	0.131*	
C48	0.5053 (7)	0.1531 (5)	0.5563 (2)	0.131 (2)	
H48A	0.5094	0.0771	0.5576	0.197*	
H48B	0.4586	0.1789	0.5817	0.197*	
H48C	0.5870	0.1818	0.5575	0.197*	

### Atomic displacement parameters (Å^2^)

**Table:**

	*U*^11^	*U*^22^	*U*^33^	*U*^12^	*U*^13^	*U*^23^
O1	0.128 (3)	0.079 (2)	0.076 (2)	0.021 (2)	0.018 (2)	−0.0026 (18)
O2	0.105 (2)	0.0805 (18)	0.0582 (16)	0.0357 (18)	0.0044 (16)	0.0046 (14)
O3	0.0700 (18)	0.085 (2)	0.095 (2)	−0.0153 (16)	0.0032 (17)	−0.0180 (18)
O4	0.077 (2)	0.166 (4)	0.135 (3)	−0.024 (3)	0.002 (3)	−0.031 (3)
C1	0.150 (6)	0.115 (4)	0.093 (4)	−0.008 (5)	0.023 (4)	0.005 (4)
C2	0.108 (4)	0.091 (3)	0.084 (3)	0.045 (3)	−0.010 (3)	0.005 (3)
C3	0.069 (2)	0.068 (2)	0.051 (2)	0.012 (2)	0.0039 (18)	−0.0084 (18)
C4	0.072 (3)	0.089 (3)	0.071 (3)	0.016 (2)	0.007 (2)	−0.015 (2)
C5	0.068 (3)	0.096 (3)	0.061 (2)	−0.005 (3)	0.020 (2)	−0.023 (2)
C6	0.065 (2)	0.081 (3)	0.0456 (18)	−0.021 (2)	0.0046 (17)	−0.0107 (19)
C7	0.084 (3)	0.118 (4)	0.052 (2)	−0.036 (3)	0.013 (2)	−0.001 (3)
C8	0.107 (4)	0.124 (4)	0.067 (3)	−0.035 (4)	−0.013 (3)	0.030 (3)
C9	0.084 (3)	0.116 (4)	0.095 (3)	−0.006 (3)	−0.011 (3)	0.044 (3)
C10	0.062 (2)	0.089 (3)	0.071 (3)	−0.005 (2)	0.003 (2)	0.018 (2)
C11	0.0539 (19)	0.063 (2)	0.0512 (19)	−0.0090 (18)	−0.0021 (16)	−0.0026 (17)
C12	0.0522 (18)	0.0567 (19)	0.0460 (17)	−0.0031 (17)	0.0052 (15)	−0.0045 (16)
C13	0.0541 (19)	0.0543 (19)	0.0518 (19)	0.0032 (16)	0.0088 (16)	0.0073 (16)
C14	0.060 (2)	0.0553 (19)	0.0438 (17)	0.0103 (17)	0.0077 (16)	0.0131 (15)
C15	0.065 (2)	0.075 (2)	0.0475 (19)	−0.001 (2)	0.0134 (18)	0.0015 (19)
C16	0.085 (3)	0.092 (3)	0.057 (2)	−0.009 (3)	0.004 (2)	−0.006 (2)
C17	0.099 (3)	0.098 (3)	0.054 (2)	0.013 (3)	0.002 (2)	−0.009 (2)
C18	0.081 (3)	0.102 (3)	0.046 (2)	0.025 (3)	0.017 (2)	0.011 (2)
C19	0.064 (2)	0.069 (2)	0.0476 (19)	0.012 (2)	0.0113 (17)	0.0142 (18)
C20	0.059 (2)	0.101 (3)	0.071 (3)	0.007 (2)	0.024 (2)	0.019 (3)
C21	0.057 (2)	0.094 (3)	0.087 (3)	−0.008 (2)	0.008 (2)	0.013 (3)
C22	0.058 (2)	0.065 (2)	0.068 (2)	−0.0024 (19)	0.0054 (19)	0.006 (2)
C23	0.105 (4)	0.100 (4)	0.133 (5)	−0.039 (4)	−0.003 (4)	−0.018 (4)
C24	0.114 (5)	0.172 (7)	0.138 (6)	0.030 (5)	−0.010 (5)	−0.038 (6)
O5	0.079 (2)	0.099 (2)	0.094 (2)	0.018 (2)	−0.0084 (19)	−0.025 (2)
O6	0.0817 (19)	0.0871 (19)	0.0662 (17)	0.0279 (17)	−0.0209 (15)	−0.0263 (15)
O7	0.152 (3)	0.0740 (18)	0.080 (2)	−0.047 (2)	0.021 (2)	−0.0224 (17)
O8	0.166 (4)	0.081 (2)	0.097 (3)	−0.035 (3)	−0.015 (3)	−0.011 (2)
C25	0.110 (4)	0.126 (5)	0.117 (5)	−0.012 (4)	−0.018 (4)	−0.011 (4)
C26	0.090 (3)	0.079 (3)	0.076 (3)	0.024 (3)	−0.023 (3)	−0.005 (2)
C27	0.064 (2)	0.058 (2)	0.0462 (18)	−0.0009 (18)	−0.0037 (17)	−0.0101 (16)
C28	0.066 (2)	0.061 (2)	0.058 (2)	0.0099 (19)	0.0024 (19)	−0.0143 (18)
C29	0.066 (2)	0.0548 (19)	0.0506 (19)	−0.0038 (18)	0.0092 (18)	−0.0154 (16)
C30	0.0551 (19)	0.0555 (18)	0.0410 (16)	−0.0080 (17)	0.0059 (15)	−0.0083 (15)
C31	0.072 (3)	0.075 (2)	0.047 (2)	−0.009 (2)	0.0035 (18)	−0.0154 (19)
C32	0.074 (3)	0.112 (4)	0.066 (3)	0.006 (3)	−0.017 (2)	−0.023 (3)
C33	0.078 (3)	0.103 (3)	0.080 (3)	0.027 (3)	−0.016 (3)	−0.023 (3)
C34	0.067 (2)	0.074 (2)	0.064 (2)	0.012 (2)	−0.002 (2)	−0.020 (2)
C35	0.0554 (19)	0.0510 (18)	0.0431 (16)	−0.0072 (16)	0.0040 (15)	−0.0099 (15)
C36	0.0543 (19)	0.0485 (17)	0.0450 (17)	−0.0034 (16)	0.0027 (15)	−0.0116 (15)
C37	0.057 (2)	0.059 (2)	0.0485 (18)	0.0048 (17)	−0.0054 (16)	−0.0164 (16)
C38	0.0490 (19)	0.084 (3)	0.0485 (19)	0.0088 (19)	−0.0069 (16)	−0.0244 (19)
C39	0.063 (2)	0.104 (3)	0.048 (2)	−0.012 (2)	0.0008 (18)	−0.020 (2)
C40	0.075 (3)	0.146 (4)	0.057 (2)	−0.030 (3)	−0.001 (2)	−0.014 (3)
C41	0.065 (3)	0.189 (6)	0.056 (3)	−0.011 (4)	0.004 (2)	−0.032 (4)
C42	0.073 (3)	0.160 (5)	0.049 (2)	0.021 (3)	−0.004 (2)	−0.043 (3)
C43	0.065 (2)	0.103 (3)	0.052 (2)	0.016 (2)	−0.0113 (19)	−0.033 (2)
C44	0.112 (4)	0.088 (3)	0.072 (3)	0.021 (3)	−0.012 (3)	−0.044 (3)
C45	0.144 (5)	0.065 (3)	0.079 (3)	−0.006 (3)	−0.006 (3)	−0.028 (2)
C46	0.102 (3)	0.066 (2)	0.060 (2)	−0.006 (2)	−0.002 (2)	−0.023 (2)
C47	0.121 (5)	0.088 (4)	0.118 (4)	−0.016 (4)	−0.006 (4)	0.007 (3)
C48	0.163 (6)	0.118 (5)	0.112 (5)	0.004 (5)	−0.019 (5)	0.025 (4)

### Geometric parameters (Å, º)

**Table:**

O1—C2	1.382 (6)	O5—C26	1.376 (5)
O1—C1	1.408 (7)	O5—C25	1.417 (6)
O2—C3	1.380 (4)	O6—C27	1.377 (4)
O2—C2	1.425 (5)	O6—C26	1.416 (5)
O3—C22	1.373 (5)	O7—C46	1.378 (5)
O3—C23	1.414 (6)	O7—C47	1.461 (6)
O4—C23	1.396 (7)	O8—C47	1.352 (6)
O4—C24	1.433 (8)	O8—C48	1.423 (6)
C1—H1A	0.9600	C25—H25A	0.9600
C1—H1B	0.9600	C25—H25B	0.9600
C1—H1C	0.9600	C25—H25C	0.9600
C2—H2A	0.9700	C26—H26A	0.9700
C2—H2B	0.9700	C26—H26B	0.9700
C3—C12	1.372 (5)	C27—C36	1.368 (5)
C3—C4	1.409 (5)	C27—C28	1.418 (5)
C4—C5	1.351 (6)	C28—C29	1.352 (5)
C4—H4A	0.9300	C28—H28A	0.9300
C5—C6	1.394 (6)	C29—C30	1.397 (5)
C5—H5A	0.9300	C29—H29A	0.9300
C6—C7	1.422 (6)	C30—C31	1.404 (5)
C6—C11	1.422 (5)	C30—C35	1.432 (4)
C7—C8	1.360 (7)	C31—C32	1.351 (6)
C7—H7A	0.9300	C31—H31A	0.9300
C8—C9	1.389 (7)	C32—C33	1.413 (6)
C8—H8A	0.9300	C32—H32A	0.9300
C9—C10	1.368 (6)	C33—C34	1.360 (6)
C9—H9A	0.9300	C33—H33A	0.9300
C10—C11	1.409 (5)	C34—C35	1.407 (5)
C10—H10A	0.9300	C34—H34A	0.9300
C11—C12	1.430 (5)	C35—C36	1.422 (5)
C12—C13	1.502 (5)	C36—C37	1.498 (4)
C13—C22	1.364 (5)	C37—C46	1.363 (5)
C13—C14	1.423 (5)	C37—C38	1.412 (5)
C14—C15	1.411 (5)	C38—C39	1.407 (6)
C14—C19	1.426 (5)	C38—C43	1.429 (5)
C15—C16	1.358 (5)	C39—C40	1.379 (6)
C15—H15A	0.9300	C39—H39A	0.9300
C16—C17	1.413 (6)	C40—C41	1.402 (7)
C16—H16A	0.9300	C40—H40A	0.9300
C17—C18	1.350 (7)	C41—C42	1.353 (8)
C17—H17A	0.9300	C41—H41A	0.9300
C18—C19	1.413 (6)	C42—C43	1.415 (7)
C18—H18A	0.9300	C42—H42A	0.9300
C19—C20	1.412 (6)	C43—C44	1.396 (7)
C20—C21	1.351 (6)	C44—C45	1.354 (7)
C20—H20A	0.9300	C44—H44A	0.9300
C21—C22	1.422 (6)	C45—C46	1.429 (5)
C21—H21A	0.9300	C45—H45A	0.9300
C23—H23A	0.9700	C47—H47A	0.9700
C23—H23B	0.9700	C47—H47B	0.9700
C24—H24A	0.9600	C48—H48A	0.9600
C24—H24B	0.9600	C48—H48B	0.9600
C24—H24C	0.9600	C48—H48C	0.9600
			
C2—O1—C1	112.3 (4)	C26—O5—C25	114.1 (4)
C3—O2—C2	118.6 (3)	C27—O6—C26	118.6 (3)
C22—O3—C23	119.5 (4)	C46—O7—C47	118.3 (4)
C23—O4—C24	113.1 (5)	C47—O8—C48	113.9 (5)
O1—C1—H1A	109.5	O5—C25—H25A	109.5
O1—C1—H1B	109.5	O5—C25—H25B	109.5
H1A—C1—H1B	109.5	H25A—C25—H25B	109.5
O1—C1—H1C	109.5	O5—C25—H25C	109.5
H1A—C1—H1C	109.5	H25A—C25—H25C	109.5
H1B—C1—H1C	109.5	H25B—C25—H25C	109.5
O1—C2—O2	112.4 (4)	O5—C26—O6	113.4 (4)
O1—C2—H2A	109.1	O5—C26—H26A	108.9
O2—C2—H2A	109.1	O6—C26—H26A	108.9
O1—C2—H2B	109.1	O5—C26—H26B	108.9
O2—C2—H2B	109.1	O6—C26—H26B	108.9
H2A—C2—H2B	107.9	H26A—C26—H26B	107.7
C12—C3—O2	116.1 (3)	C36—C27—O6	116.2 (3)
C12—C3—C4	120.9 (4)	C36—C27—C28	121.2 (3)
O2—C3—C4	123.0 (4)	O6—C27—C28	122.7 (3)
C5—C4—C3	119.9 (4)	C29—C28—C27	120.0 (3)
C5—C4—H4A	120.0	C29—C28—H28A	120.0
C3—C4—H4A	120.0	C27—C28—H28A	120.0
C4—C5—C6	122.1 (4)	C28—C29—C30	121.8 (3)
C4—C5—H5A	119.0	C28—C29—H29A	119.1
C6—C5—H5A	119.0	C30—C29—H29A	119.1
C5—C6—C7	122.8 (4)	C29—C30—C31	122.6 (3)
C5—C6—C11	118.7 (4)	C29—C30—C35	118.3 (3)
C7—C6—C11	118.5 (4)	C31—C30—C35	119.1 (3)
C8—C7—C6	120.7 (4)	C32—C31—C30	121.6 (4)
C8—C7—H7A	119.7	C32—C31—H31A	119.2
C6—C7—H7A	119.7	C30—C31—H31A	119.2
C7—C8—C9	120.8 (5)	C31—C32—C33	119.5 (4)
C7—C8—H8A	119.6	C31—C32—H32A	120.2
C9—C8—H8A	119.6	C33—C32—H32A	120.2
C10—C9—C8	120.4 (5)	C34—C33—C32	120.7 (4)
C10—C9—H9A	119.8	C34—C33—H33A	119.7
C8—C9—H9A	119.8	C32—C33—H33A	119.7
C9—C10—C11	121.0 (4)	C33—C34—C35	121.2 (4)
C9—C10—H10A	119.5	C33—C34—H34A	119.4
C11—C10—H10A	119.5	C35—C34—H34A	119.4
C10—C11—C6	118.7 (4)	C34—C35—C36	122.3 (3)
C10—C11—C12	122.2 (3)	C34—C35—C30	117.9 (3)
C6—C11—C12	119.1 (4)	C36—C35—C30	119.9 (3)
C3—C12—C11	119.3 (3)	C27—C36—C35	118.9 (3)
C3—C12—C13	120.4 (3)	C27—C36—C37	119.5 (3)
C11—C12—C13	120.2 (3)	C35—C36—C37	121.6 (3)
C22—C13—C14	120.3 (3)	C46—C37—C38	118.9 (3)
C22—C13—C12	120.2 (3)	C46—C37—C36	119.2 (3)
C14—C13—C12	119.5 (3)	C38—C37—C36	121.9 (3)
C15—C14—C13	122.9 (3)	C39—C38—C37	121.9 (3)
C15—C14—C19	118.0 (3)	C39—C38—C43	118.1 (4)
C13—C14—C19	119.1 (3)	C37—C38—C43	120.0 (4)
C16—C15—C14	121.5 (4)	C40—C39—C38	121.1 (4)
C16—C15—H15A	119.3	C40—C39—H39A	119.5
C14—C15—H15A	119.3	C38—C39—H39A	119.5
C15—C16—C17	120.1 (4)	C39—C40—C41	120.2 (5)
C15—C16—H16A	119.9	C39—C40—H40A	119.9
C17—C16—H16A	119.9	C41—C40—H40A	119.9
C18—C17—C16	120.2 (4)	C42—C41—C40	120.3 (5)
C18—C17—H17A	119.9	C42—C41—H41A	119.8
C16—C17—H17A	119.9	C40—C41—H41A	119.8
C17—C18—C19	121.2 (4)	C41—C42—C43	121.1 (5)
C17—C18—H18A	119.4	C41—C42—H42A	119.5
C19—C18—H18A	119.4	C43—C42—H42A	119.5
C20—C19—C18	122.6 (4)	C44—C43—C42	122.3 (4)
C20—C19—C14	118.4 (4)	C44—C43—C38	118.5 (4)
C18—C19—C14	119.0 (4)	C42—C43—C38	119.1 (5)
C21—C20—C19	121.7 (4)	C45—C44—C43	121.8 (4)
C21—C20—H20A	119.2	C45—C44—H44A	119.1
C19—C20—H20A	119.2	C43—C44—H44A	119.1
C20—C21—C22	120.0 (4)	C44—C45—C46	119.2 (4)
C20—C21—H21A	120.0	C44—C45—H45A	120.4
C22—C21—H21A	120.0	C46—C45—H45A	120.4
C13—C22—O3	116.8 (3)	C37—C46—O7	116.7 (3)
C13—C22—C21	120.5 (4)	C37—C46—C45	121.5 (4)
O3—C22—C21	122.7 (4)	O7—C46—C45	121.8 (4)
O4—C23—O3	112.7 (4)	O8—C47—O7	109.2 (5)
O4—C23—H23A	109.1	O8—C47—H47A	109.8
O3—C23—H23A	109.1	O7—C47—H47A	109.8
O4—C23—H23B	109.1	O8—C47—H47B	109.8
O3—C23—H23B	109.1	O7—C47—H47B	109.8
H23A—C23—H23B	107.8	H47A—C47—H47B	108.3
O4—C24—H24A	109.5	O8—C48—H48A	109.5
O4—C24—H24B	109.5	O8—C48—H48B	109.5
H24A—C24—H24B	109.5	H48A—C48—H48B	109.5
O4—C24—H24C	109.5	O8—C48—H48C	109.5
H24A—C24—H24C	109.5	H48A—C48—H48C	109.5
H24B—C24—H24C	109.5	H48B—C48—H48C	109.5
			
C1—O1—C2—O2	−68.1 (6)	C25—O5—C26—O6	−73.6 (5)
C3—O2—C2—O1	−64.4 (6)	C27—O6—C26—O5	−71.5 (5)
C2—O2—C3—C12	157.1 (4)	C26—O6—C27—C36	−174.9 (4)
C2—O2—C3—C4	−25.7 (6)	C26—O6—C27—C28	5.5 (6)
C12—C3—C4—C5	0.2 (7)	C36—C27—C28—C29	−1.0 (6)
O2—C3—C4—C5	−176.9 (4)	O6—C27—C28—C29	178.5 (4)
C3—C4—C5—C6	1.0 (7)	C27—C28—C29—C30	0.3 (6)
C4—C5—C6—C7	177.2 (4)	C28—C29—C30—C31	179.4 (4)
C4—C5—C6—C11	−1.1 (6)	C28—C29—C30—C35	0.6 (5)
C5—C6—C7—C8	−178.7 (4)	C29—C30—C31—C32	−177.9 (4)
C11—C6—C7—C8	−0.3 (6)	C35—C30—C31—C32	0.9 (6)
C6—C7—C8—C9	0.3 (8)	C30—C31—C32—C33	−0.4 (7)
C7—C8—C9—C10	−0.2 (9)	C31—C32—C33—C34	−0.4 (8)
C8—C9—C10—C11	0.0 (8)	C32—C33—C34—C35	0.7 (7)
C9—C10—C11—C6	0.0 (6)	C33—C34—C35—C36	178.9 (4)
C9—C10—C11—C12	178.4 (4)	C33—C34—C35—C30	−0.3 (6)
C5—C6—C11—C10	178.6 (4)	C29—C30—C35—C34	178.3 (3)
C7—C6—C11—C10	0.2 (5)	C31—C30—C35—C34	−0.5 (5)
C5—C6—C11—C12	0.1 (5)	C29—C30—C35—C36	−0.8 (5)
C7—C6—C11—C12	−178.3 (3)	C31—C30—C35—C36	−179.7 (3)
O2—C3—C12—C11	176.1 (3)	O6—C27—C36—C35	−178.8 (3)
C4—C3—C12—C11	−1.2 (6)	C28—C27—C36—C35	0.8 (5)
O2—C3—C12—C13	−3.0 (5)	O6—C27—C36—C37	1.1 (5)
C4—C3—C12—C13	179.7 (4)	C28—C27—C36—C37	−179.3 (3)
C10—C11—C12—C3	−177.4 (4)	C34—C35—C36—C27	−179.0 (3)
C6—C11—C12—C3	1.0 (5)	C30—C35—C36—C27	0.1 (5)
C10—C11—C12—C13	1.7 (5)	C34—C35—C36—C37	1.1 (5)
C6—C11—C12—C13	−179.9 (3)	C30—C35—C36—C37	−179.8 (3)
C3—C12—C13—C22	−97.9 (4)	C27—C36—C37—C46	−89.5 (5)
C11—C12—C13—C22	83.1 (4)	C35—C36—C37—C46	90.4 (5)
C3—C12—C13—C14	82.9 (4)	C27—C36—C37—C38	89.4 (4)
C11—C12—C13—C14	−96.1 (4)	C35—C36—C37—C38	−90.7 (4)
C22—C13—C14—C15	−179.1 (3)	C46—C37—C38—C39	175.6 (4)
C12—C13—C14—C15	0.1 (5)	C36—C37—C38—C39	−3.3 (5)
C22—C13—C14—C19	0.1 (5)	C46—C37—C38—C43	−2.7 (6)
C12—C13—C14—C19	179.3 (3)	C36—C37—C38—C43	178.4 (3)
C13—C14—C15—C16	179.4 (4)	C37—C38—C39—C40	−179.6 (4)
C19—C14—C15—C16	0.2 (5)	C43—C38—C39—C40	−1.2 (6)
C14—C15—C16—C17	−0.8 (6)	C38—C39—C40—C41	−0.8 (7)
C15—C16—C17—C18	1.1 (7)	C39—C40—C41—C42	2.3 (8)
C16—C17—C18—C19	−0.7 (7)	C40—C41—C42—C43	−1.6 (8)
C17—C18—C19—C20	−179.5 (4)	C41—C42—C43—C44	179.3 (5)
C17—C18—C19—C14	0.1 (6)	C41—C42—C43—C38	−0.4 (7)
C15—C14—C19—C20	179.8 (3)	C39—C38—C43—C44	−177.9 (4)
C13—C14—C19—C20	0.6 (5)	C37—C38—C43—C44	0.5 (6)
C15—C14—C19—C18	0.2 (5)	C39—C38—C43—C42	1.8 (6)
C13—C14—C19—C18	−179.0 (3)	C37—C38—C43—C42	−179.8 (4)
C18—C19—C20—C21	179.3 (4)	C42—C43—C44—C45	−178.9 (5)
C14—C19—C20—C21	−0.3 (6)	C38—C43—C44—C45	0.8 (7)
C19—C20—C21—C22	−0.6 (7)	C43—C44—C45—C46	0.1 (8)
C14—C13—C22—O3	179.7 (3)	C38—C37—C46—O7	−178.9 (4)
C12—C13—C22—O3	0.5 (5)	C36—C37—C46—O7	0.0 (6)
C14—C13—C22—C21	−1.0 (6)	C38—C37—C46—C45	3.7 (6)
C12—C13—C22—C21	179.8 (3)	C36—C37—C46—C45	−177.4 (4)
C23—O3—C22—C13	175.1 (4)	C47—O7—C46—C37	148.1 (4)
C23—O3—C22—C21	−4.1 (6)	C47—O7—C46—C45	−34.5 (7)
C20—C21—C22—C13	1.3 (6)	C44—C45—C46—C37	−2.4 (8)
C20—C21—C22—O3	−179.5 (4)	C44—C45—C46—O7	−179.7 (5)
C24—O4—C23—O3	64.4 (7)	C48—O8—C47—O7	65.8 (7)
C22—O3—C23—O4	67.9 (7)	C46—O7—C47—O8	81.5 (6)

### Hydrogen-bond geometry (Å, º)

**Table:**

*D*—H···*A*	*D*—H	H···*A*	*D*···*A*	*D*—H···*A*
C34—H34*A*···O8^i^	0.93	2.40	3.251 (5)	152
C39—H39*A*···O5^ii^	0.93	2.52	3.417 (5)	161

Symmetry codes: (i) *x*+1/2, −*y*+1/2, −*z*+1; (ii) *x*+1/2, −*y*+3/2, −*z*+1.

## References

[bb1] Bruker (2004). *APEX2, *SAINT** and *SADABS* Bruker AXS Inc., Madison, Wisconsin,USA.

[bb2] Brunel, J. M. (2006). *Chem. Rev.* **105**, 857–897. 10.1021/cr040079g 15755079

[bb3] Sheldrick, G. M. (2008). *Acta Cryst.* A**64**, 112–122. 10.1107/S0108767307043930 18156677

[bb4] Shi, M. & Wang, C.-J. (2002). *Tetrahedron Asymmetry*, **13**, 2161–2166.

[bb5] Tachi, Y., Nakayama, S., Tani, F., Ueno, G. & Naruta, Y. (1999). *Acta Cryst.* C**55**, 1351–1353.

[bb6] Westrip, S. P. (2010). *J. Appl. Cryst.* **43**, 920–925.

[bb7] Zong, H., Huang, H.-Y., Hu, B., Bian, G.-L. & Song, L. (2011). *Acta Cryst.* E**67**, o222. 10.1107/S1600536810053018 PMC3050160 21522721

